# Efficacy and Safety of a Single Dose of Ivermectin, Diethylcarbamazine, and Albendazole for Treatment of Lymphatic Filariasis in Côte d’Ivoire: An Open-label Randomized Controlled Trial

**DOI:** 10.1093/cid/ciz1050

**Published:** 2019-10-23

**Authors:** Catherine M Bjerum, Allassane F Ouattara, Méité Aboulaye, Olivier Kouadio, Vanga K Marius, Britt J Andersen, Gary J Weil, Benjamin G Koudou, Christopher L King

**Affiliations:** 1 Center for Global Health and Diseases, Case Western Reserve University School of Medicine, Cleveland, Ohio, USA; 2 Centre Suisse de Recherche Scientifique en Côte d’Ivoire, Abidjan, Côte d’Ivoire; 3 Université Nangui Abrogoua, Abidjan, Côte d’Ivoire; 4 Programme National de la Lutte Contre la Schistosomiase, Les Geohelminthiases et la Filariose Lymphatique, Abidjan, Côte d’Ivoire; 5 Universite Alassane Ouattara Centre Hospitalier Universitaire de Bouake, Bouaké, Côte d’Ivoire; 6 Infectious Diseases Division, Department of Medicine, Washington University School of Medicine, St Louis, Missouri, USA; 7 Veterans Affairs Research Service, Cleveland Veterans Affairs Medical Center, Cleveland, Ohio, USA

**Keywords:** lymphatic filariasis, ivermectin, diethylcarbamazine, albendazole, efficacy

## Abstract

**Background:**

Improved drug regimens are needed to accelerate elimination of lymphatic filariasis in Africa. This study determined whether a single co-administered dose of ivermectin plus diethylcarbamazine plus albendazole [IDA] is noninferior to standard 3 annual doses of ivermectin plus albendazole (IA) used in many LF-endemic areas of Africa.

**Methods:**

Treatment-naive adults with *Wuchereria bancrofti* microfilaremia in Côte d’Ivoire were randomized to receive a single dose of IDA (n = 43) or 3 annual doses of IA (n = 52) in an open-label, single-blinded trial. The primary endpoint was the proportion of participants who were microfilaria (Mf) negative at 36 months. Secondary endpoints were Mf clearance at 6, 12, and 24 months; inactivation of adult worm nests; and safety.

**Results:**

At 36 months posttreatment with IDA, 18/33 (55%; 95% CI, 38–72%) cleared Mf versus 33/42 (79%; 67–91%) with IA (*P* = .045). At 6 and 12 months IDA was superior to IA in clearing Mf (89% [77–99%] and 71% [56–85%]), respectively, versus 34% (20–48%) and 26% (14–42%) (*P* < .001). IDA was equivalent to IA at 24 months (61% [45–77%] vs 54% [38–72%]; *P* = .53). IDA was superior to IA for inactivating adult worms at all time points. Both treatments were well tolerated, and there were no serious adverse events.

**Conclusions:**

A single dose of IDA was superior to 2 doses of IA in reducing the overall Mf burden by 24 months. Reinfection may have contributed to the lack of sustained clearance of Mf with IDA.

**Clinical Trials Registration:**

NCT02974049.

## Teaser

A single dose of ivermectin, diethylcarbamazine, and albendazole is superior to standard treatment with ivermectin and albendazole for microfilarial clearance at 6 and 12 months and equivalent to 2 annual doses of ivermectin and albendazole at 24 months.

Lymphatic filariasis (LF) caused by *Wuchereria bancrofti*, *Brugia malayi*, or *Brugia timori* is a widespread mosquito-borne parasitic disease caused by nematode parasites. Lymphatic filariasis can result in significant deformity and chronic disability, due to lymphedema, elephantiasis, and hydroceles. The World Health Organization (WHO) has targeted LF for global elimination as a public health problem. Efforts are largely based on a mass drug administration (MDA) approach utilizing 1 of 3 antifilarial drug regimens: (1) ivermectin (IVM) plus albendazole (ALB) in regions of Africa where *Onchocerca volvulus* is coendemic, (2) ALB alone in areas where LF is coendemic with *Loa loa* and MDA with IVM is not in use, or (3) ALB plus diethylcarbamazine (DEC) in LF-endemic areas outside Africa and in regions of Africa where *Loa loa* and *O. volvulus* are not endemic [1]. Because of the limited efficacy of existing drug combinations, 5 or more annual rounds of treatment with high compliance are required to meet WHO targets and interrupt transmission [2]. Consistently achieving such high coverage with repeated rounds of treatment has been difficult in many settings. Recently, we reported that a single coadministered dose of IVM plus ALB plus DEC (referred to as IDA) in Papua New Guinea (PNG) resulted in sustained clearance of microfilaria (Mf) in 96% of individuals with moderate to heavy infections of *W. bancrofti* for up to 3 years [3, 4] and is well tolerated [5]. Consequently, WHO changed its guidelines in 2017 to recommend IDA for MDA in areas outside of sub-Saharan Africa that are not likely to achieve elimination goals by 2020 [6]. In Africa, IDA has not been tested in comparison to the standard treatment of IVM plus ALB (referred to as IA).

The use of IDA would be of particular value in LF-endemic areas in Africa that are nonendemic for onchocerciasis or loiasis. The purpose of this study is to test whether a single dose of IDA can sustain complete suppression of Mf and whether IDA is noninferior to the standard therapy of annual treatment with IA for up to 3 years. Even if clearance was 15% less effective at 2 or 3 years compared with the standard treatment, a single dose compared with multiple dosing would provide a marked advantage.

## METHODS

### Study Population and Ethical Approval

Participants were recruited from Agboville District, Côte d’Ivoire, which is endemic for LF and nonendemic for onchocerciasis or loiasis. Individuals aged 18–70 years with 50 or more Mf per milliliter but otherwise healthy and having no treatment with ALB or IVM within the past 5 years were eligible. Exclusion criteria were a positive pregnancy test; a history of chronic kidney or liver disease; a serum alanine transaminase, aspartate transaminase, or creatinine level more than 1.5 times the upper limits of normal; or severe anemia (blood hemoglobin <7 g/dL). Persons with onchocerciasis were excluded. The study protocol and related documents were approved by institutional review boards in Cleveland, Ohio (University Hospitals of Cleveland Medical Center Institutional Review Board no. 08-14-13) and in Côte d’Ivoire (Comité National d’Ethique et de la Recherche [CNER] no. 008/MSLS/CNER/-kp). The study was registered with ClinicalTrials.gov (NCT02974049). Fourteen participants in the IDA treatment arm who were microfilaremic 24 months after treatment were retreated at that time, based on recommendation of the Ivorian CNER. Written informed consent was obtained from all individuals prior to enrollment.

### Study Design, Randomization, and Interventions

**Figure 1.**
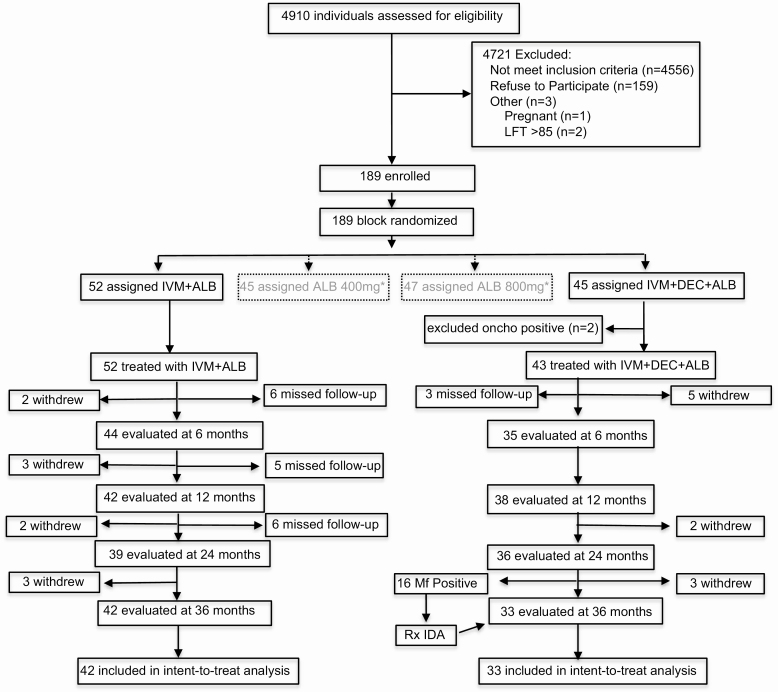
Trial profile. Abbreviations: ALB, albendazole; DEC, diethylcarbamazine; IDA, ivermectin plus diethylcarbamazine plus albendazole; IVM, ivermectin; LFT, liver function tests, aspartate aminotransferase and alanine aminotransferase with one or both >85 international unit representing >1.5 fold above normal values; Mf, microfilaria; oncho, *Onchocerca volvulus*; Rx, treatment. * The albendazole alone arms are part of a different study.

This was a randomized, parallel-group, open-label trial (Figure 1). Two studies were conducted at the same time using the same IA control arm because of the challenge of finding sufficient eligible subjects. The second study assessed the use of ALB alone for areas coendemic for loiasis and is being reported separately. Eligible individuals were randomized to 1 of 4 treatment arms (blocks of 4, randomized 1:1:1:1), with 2 arms reported here as follows: Arm 1, 3 annual doses of coadministered IVM 200 µg/kg (Merck & Co.) plus ALB 400 mg (GlaxoSmithKline); Arm 2, IVM 200 µg/kg plus DEC 6 mg/kg (Sanofi SA) plus ALB 400 mg provided only once at the beginning of the study. Investigators and staff who evaluated adverse events (AEs), performed ultrasound examinations, or read slides for Mf were masked with respect to treatment arm assignments. Withdrawals included individuals that moved from the area, 3 in the IA arm and 4 in IDA arm, or who did not wish to have further venous night blood draws.

### Safety Monitoring

After directly observed treatment with study medications, participants were assessed at 24 hours after treatment, then passively monitored on days 2 through 7 posttreatment. Adverse events were scored using a modified version of the National Cancer Institute Common Terminology Criteria for Adverse Events, version 4.0.

### Parasitology Determination

We screened for *W. bancrofti* infection using the rapid test for circulating filarial antigenemia (CFA; Filariasis Test Strip or FTS; Alere, Inc) [7]. Positive tests were scored semiquantitatively as previously described [8]. FTS-positive individuals were tested for Mf by membrane filtration (5 µM; Nuclepore Corporation) using 2 separate 1-mL aliquots of venous blood collected between 2130 and 0100 hours [9]. Two microscopists independently read Giemsa-stained filters. FTS and Mf filtration were repeated at 6, 12, 24, and 36 months posttreatment. The presence of onchocerciasis was evaluated by skin snips taken from the iliac crests and by testing for antibodies to a recombinant *O. volvulus* antigen (Ov16 Rapid Test; Standard Diagnostics, Inc) [10]. CFA levels were measured on a subset of participants at baseline and at 12 months by enzyme-linked immunosorbent assay [11].

Scrotal ultrasound examinations were performed on men using a SonoScape S8 portable ultrasound system, equipped with a 5- to 10-MHz linear array transducer (International Diagnostic Devices, Inc). Color and pulsed-wave Doppler modes were used to differentiate lymphatic vessels from blood vessels. Adult worm nests were identified based on movement of worms (the “filarial dance sign”) [12] and findings digitally recorded. Only participants with worm nests at baseline were rescanned at 6, 12, 24, and 36 months posttreatment, with the location of worm nests mapped and validated by review of recorded images. Worm nest number, location, and maximum diameter were recorded along with the degree of lymphatic vessel dilatation.

### Statistical Analysis

The primary outcome, that a single dose of IDA was noninferior for complete clearance of Mf to 3 annual doses of standard IA treatment at 36 months, was evaluated with a noninferiority margin of 15% based on variance of IA for complete clearance of Mf at 12 and/or 24 months posttreatment from prior studies [13–18]. We also tested Mf clearance rates between the 2 groups at 6, 12, 24, and 36 months using a 2-sided Fisher’s exact test and estimated relative risk of IDA relative to IA. To compare Mf counts across time and between treatment arms, we used a negative binominal generalized estimating equation model (R-Project version 3.5.1). The model included follow-up time points at 6, 12, and 24 months, with the interaction between treatment and time as fixed effects adjusted for age, sex, and multiple comparisons. Microfilarial levels at 36 months could not be evaluated because Mf-positive individuals in the IDA arm were retreated at 24 months. An intention-to-treat (ITT) analysis was performed for all individuals for whom a sample was collected at 36 months for the primary outcome. On the basis of published data using multiple doses of IA and single-dose IDA, predicting a 30% drop-out rate by 36 months, we would need 70 participants per group with a power of 80% to detect the noninferiority margin of 15%. Participants in the IDA arm who were retreated because of microfilarmia at 24 months were considered to be microfilaremic at the 36-month time point.

## RESULTS

### Enrollment, Treatment Assignment, and Baseline Filariasis Test Results

A total of 189 participants were randomized to 1 of 4 treatment arms, 97 of whom were randomized to treatment with IA or IDA (Figure 1). Participants were enrolled between 5 February 2015 and 13 September 2015. The drop-out rate was 22.7%. Eighty-four (88%) of the participants were men, which reflects a higher infection rate in males and the reluctance of women to participate in night blood sampling (Table 1). Pretreatment Mf counts ranged from 51 to 1498 Mf/mL, with a geometric mean of 192 Mf/mL, and were equivalent between the 2 groups.

### Effects of Treatment on Microfilaremia

With a single dose of IDA 89%, 71%, 61%, and 55% of participants were free of Mf at 6, 12, 24, and 36 months, respectively (Table 2). Participants who were still microfilaremic at 24 months were retreated with IDA. Retreated individuals in the triple-drug arm at 24 months and seen at 36 months were considered to be microfilaremic, since individuals remain microfilaremic without treatment. Treatment with IA administered once per year for 3 years resulted in 34%, 26%, 54%, and 79% of participants who were Mf free at 6, 12, 24, and 36 months, respectively (Table 2). Three annual doses of IA were better than a single dose of IDA for Mf clearance at 36 months (24 percentage points difference, which was greater than the noninferiority margin of 15%; *P* = .04). IDA clearance rates at 6 and 12 months were significantly greater relative to IA (improvement of 83% and 61%, respectively) (Table 2) and showed a trend for greater Mf clearance at 24 months, but this difference was not significant (*P* = .53).

**Table 2.** Clearance of Microfilaremia After Treatment for Lymphatic Filariasis

	Months After Initial Treatment			
	6	12	24	36
Triple-drug regimen administered once				
** **Participants, n	35	38	36	33
** **Participants with complete clearance of Mf, n	31	27	22	18
** **Clearance, % (95% CI)^a^	89 (77–99)	71 (56–85)	61 (45–77)	55 (38–72)^b^
Two-drug regimen administered annually for 3 years				
** **Participants, n	44	42	39	42
** **Participants with complete clearance of Mf, n	15	11	21	33
** **Clearance, % (95% CI)^a^	34 (20–48)	26 (14–42)	54 (38–70)	79 (67–91)
** ** *P* value^c^	<.001	<.001	.53	.045
** **Relative risk of incomplete^d^ clearance (95% CI)	0.17 (.06–.45)	0.39 (.23–.67)	0.84 (.50- 1.43)	2.14 (1.06–4.23)
** ** *P* value^e^	<.001	<.001	.64	.033

Abbreviations: CI, confidence interval; Mf, microfilaria.

^a^The 95% CIs were calculated using the binomial exact methods.

^b^Participants who were Mf positive at 24 months and retreated were assumed to still be Mf positive at 36 months for calculating clearance rates.

^c^ *P* values derived by using chi-square test between the 3-drug regimen compared with the 2-drug arm.

^d^Relative risks are shown for the arm that received the 3-drug regimen as compared with the arm that received the 2-drug regimen.

^e^ *P* values are for the relative risks between the 2 treatment groups.

**Figure 2.**
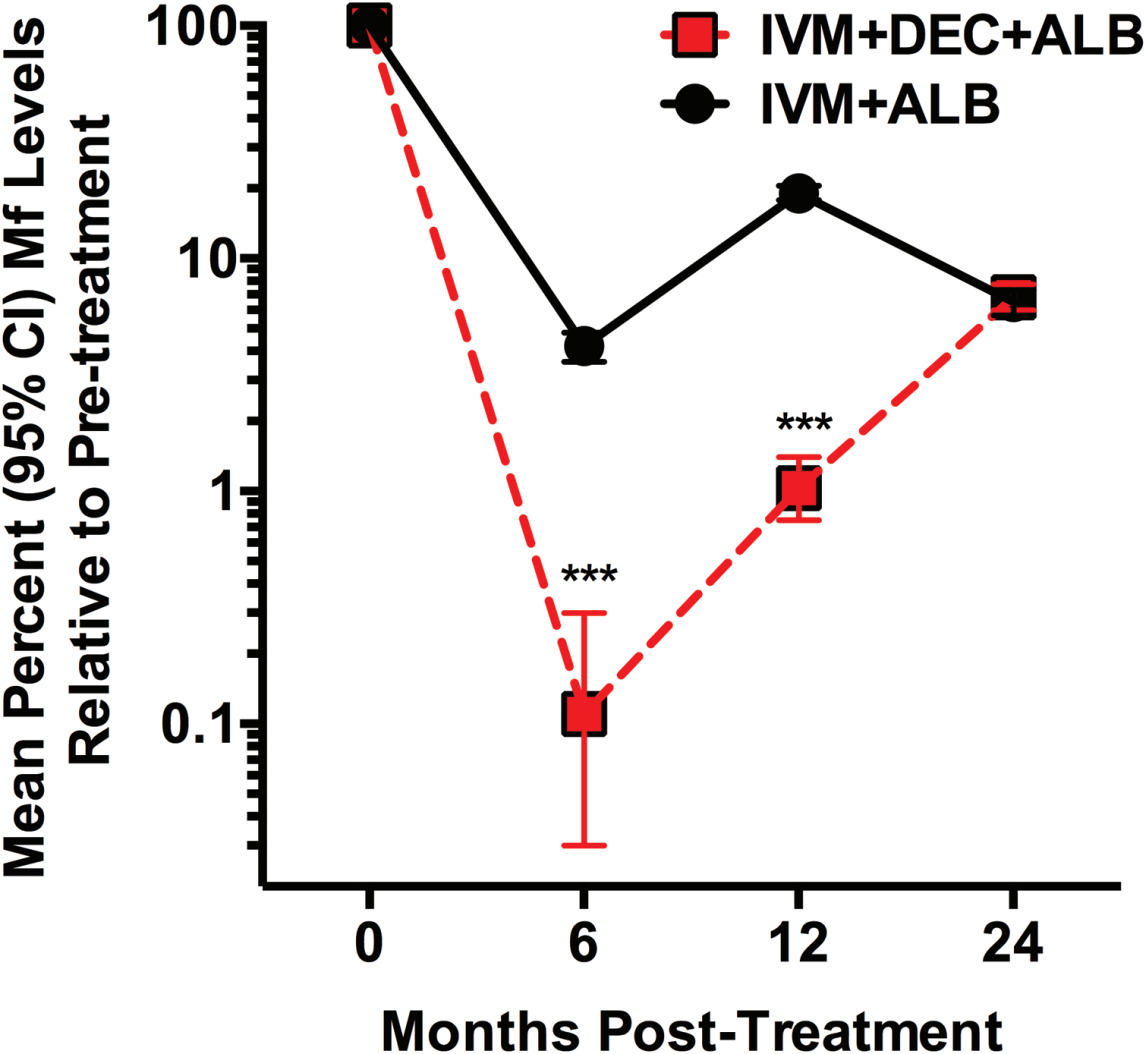
Mean percent decrease (95% CI) in microfilaremia levels at 6, 12, and 24 months after treatment for the 2 treatment arms, as determined by microfilaria (Mf) counts at each time point divided by baseline Mf count for each participant. The numbers of participants for each time point are shown in Table 2. Participants without detectable Mf are included in the analysis and considered to have 100% clearance. ***P < .001 using Mann–Whitney test (2-sided) comparing individuals at 6 and 12 months. Abbreviations: ALB, albendazole; CI, confidence interval; DEC, diethylcarbamazine; IVM, ivermectin; Mf, microfilaria.

Compared with baseline, IDA reduced mean individual Mf levels by more than 99% at 6 months, 98.9% at 12 months, and 93.2% at 24 months (Figure 2). Estimates of Mf counts for this analysis could not be calculated at 36 months because individuals who were Mf positive at 24 months were retreated. Annual IA treatment reduced mean individual Mf levels by 95.8% at 6 months, 80.1% at 12 months, 93.5% at 24 months, and 93.7% at 36 months. Reductions in Mf levels were significantly greater at 6 and 12 months after IDA (*P* < .001) (Figure 2), but not at 24 months. Overall, there was an 81% greater reduction in Mf levels at 12 months compared with IA (incidence rate ratio [IRR], .19; 95% confidence interval [CI], .12–.33; *P* < .0001), but no benefit of IDA treatment on reduction in Mf levels at 24 months (IRR, .89; 95% CI, .58–1.37; *P* = .59). The cumulative Mf burden following a single dose of IDA over 24 months was an average of 7.2 Mf/mL (783/109; total Mf at 6, 12, and 24 months divided by the total number of individuals examined at the same time points) versus 27.4 Mf/mL (3431/125) over the same period after 2 doses of IA, representing a 3.8-fold reduction in Mf levels with single-dose IDA.

### Effects of Treatment on Circulating Filarial Antigenemia

**Figure 3.**
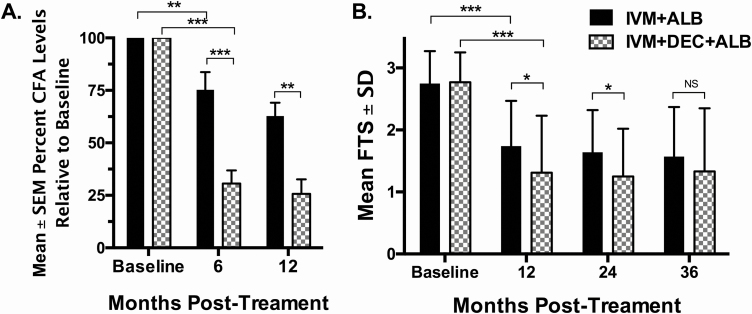
Changes in circulating filarial antigen (CFA) relative to baseline as determined by enzyme-linked immunosorbent assay (*A*) or Filariasis Test Strip (FTS) score (*B*). *A*, The mean (±standard error of the mean [SEM]) percentage of persisting CFA levels relative to baseline (CFA levels at 6 or 12 months/CFA levels at baseline) is shown (ivermectin plus albendazole [IA] arm: n = 30, 28, and 15 at baseline and 6 and 12 months, respectively; ivermectin plus diethylcarbamazine plus albendazole [IDA] arm: n = 26, 24, and 14 at the same time points). *B*, Mean (±SD) FTS score results for all individuals for which a microfilarial level was determined. *P < .05, **P < .01, ***P < .001 using Mann–Whitney *U* test for comparisons between IA and IDA groups. Abbreviations: ALB, albendazole; CFA, circulating filarial antigen; DEC, diethylcarbamazine; FTS, Filariasis Test Strip; IA, ivermectin plus albendazole; IDA, ivermectin plus diethylcarbamazine plus albendazole; IVM, ivermectin; SD, standard deviation; SEM, standard error of the mean.

Pretreatment mean levels of CFA were similar between the treatment groups (Table 1). The CFA levels decreased to a much greater extent in the arm that received IDA at 6 and 12 months (Figure 3A) (*P* < .01). For both treatment arms, there was no further significant reduction in CFA levels at 24 and 36 months compared with the 12-month time point. This lack of change in mean CFA in the IDA arm from 24 to 36 months is notable in that 16 participants who were Mf positive at 24 months were retreated with IDA (see below).

**Table 1.** Baseline Characteristics of the Study Participants

Characteristics	IA (n = 52)	IDA (n = 43)
Age, median (IQR), years	35 (26, 45)	35 (25, 47)
Male sex, n (%)	46 (88)	38 (88)
Microfilaria count, geometric mean (range), Mf/mL	190 (51–1064)	198 (51–1498)
Men with worm nests/number examined (%)	34/41 (83)	22/32 (69)
Mean worm nests^a^ (range)	2.5 (1-6)	2.7 (1-7)
Mean ± SD FTS^b^	2.75 ± 0.52	2.74 ± 0.48
Circulating antigen, geometric mean (95% CI),^c^ ng/mL	25.6 (17.4–33.8)	35.9 (22.3–49.5)

Abbreviations: CI, confidence interval; ELISA, enzyme-linked immunosorbent assay; FTS, Filarial Test Strip; IA, ivermectin plus albendazole; IDA, ivermectin plus diethylcarbamazine plus albendazole; IQR, interquartile range; Mf, microfilaria; SD, standard deviation.

^a^In men with 1 or more worm nests detected by ultrasound.

^b^Average of FTS score of 1, 2, or 3.

^c^Measured by ELISA using the same antibodies used for the FTS.

### Effects of Treatment on Filarial Adult Worm Nests

Fifty-six of 73 men (77%) who had scrotal ultrasounds at baseline had nests with motile adult worms: 22 men in the IDA arm and 34 men in the IA arm, with an average of 2.6 (range, 1–7) nests (Table 1). IDA treatment inactivated all detectable worm nests in 74%, 81%, 79%, and 79% of participants at 6, 12, 24, and 36 months, respectively (Table 3). Worms that were inactivated at 12 months posttreatment remained inactive for the duration of the study. IA treatment inactivated all detectable worm nests noted at baseline in 18%, 36%, 44%, and 40% of participants at 6, 12, 24, and 36 months, respectively.

**Table 3.** Participants With Inactivation of All Detectable Worm Nests After Treatment

	Months After Initial Treatment			
	6	12	24	36
IDA administered once				
Participants with worm nests examined,^a^ n	19	21	19	19
Participants with complete inactivation of worm nests, n	14	17	15	15
% (95% CI)	74 (54–94)	81 (64–97)	79 (61–97)	79 (61–97)
IA administered annually for 3 years				
** **Participants with worm nests,^a^ n	28	28	27	25
** **Participants with complete inactivation of worm nests, n (%)	5	10	12	10
** **% (95% CI)	18 (4–32)	36 (18–54)	44 (25–63)	40 (22–58)
** ** *P* value (chi-square)	<.001	.002	.08	.032

Abbreviations: CI, confidence interval; IA, ivermectin plus albendazole; IDA, ivermectin plus diethylcarbamazine plus albendazole.

^a^Only data from participants with worm nests detected by ultrasound at baseline are included in this table.

**Figure 4.**
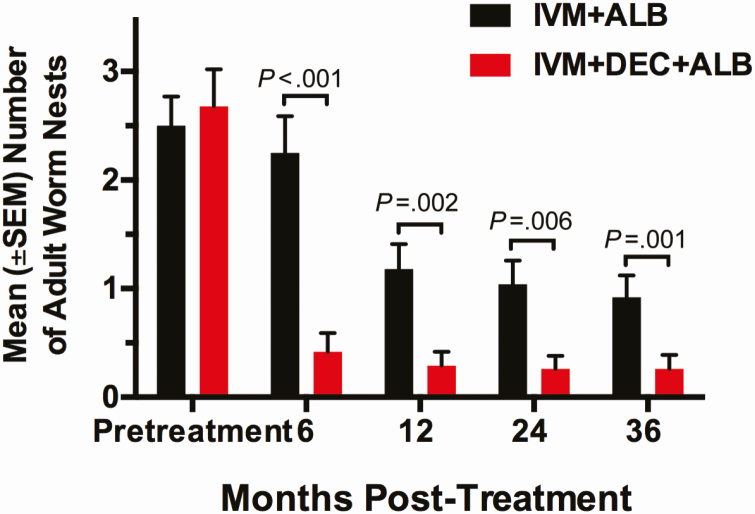
The effect of treatment on the number of adult worm nests detected by ultrasonography. The mean (±SEM) number of worm nests in scrotal lymphatics pretreatment and at 6, 12, 24, and 36 months posttreatment is shown. Only men with detectable worm nests in the scrotal lymphatics at baseline were examined at follow-up. Annual ivermectin plus albendazole (IA) treatment significantly reduced worm nests from baseline at 12, 24, and 36 months (*P* = .015, *P* < .001, and *P* < .001; paired Wilcoxon rank sum test). A single treatment with ivermectin plus diethylcarbamazine plus albendazole (IDA) resulted in significantly greater reductions in worm nest counts relative to annual treatment with IA for all time points posttreatment (*P* < .001). The significance of this difference was determined by the Mann–Whitney *U* test. The 5 participants in the IDA arm who were retreated with IDA at 24 months were included in the worm nest analysis, because the additional round of treatment had almost no additional effect on worm nests (see text). Abbreviations: ALB, albendazole; DEC, diethylcarbamazine; IA, ivermectin plus albendazole; IDA, ivermectin plus diethylcarbamazine plus albendazole; IVM, ivermectin; SEM, standard error of the mean.

Relative to baseline, IDA treatment reduced the mean number of detectable worm nests in men by 84%, 89%, 90%, and 90% at 6, 12, 24, and 36 months, respectively (Figure 4). IA resulted in a lower reduction in worm nests at the same time points of 10%, 53%, 60%, and 63%, respectively. Worm nest size and the degree of lymphatic vessel dilatation did not change at any time point in either arm. We retreated 5 individuals in the IDA arm who were microfilaremic and had persisting worm nests at 24 months: 4 participants had a single worm nest and 1 participant had 2 nests. One year after retreatment with IDA, 4 of 5 individuals retained the same number of worm nests and 1 man with a single worm nest showed inactivation.

### Appearance of New Worm Nests After Treatment

Some study participants acquired new worm nests (Supplementary Figure 1). Seven participants in the IA treatment arm had 1 or more new adult worm nests at 6 months, with a total of 15 new worm nests after treatment. Four additional IA recipients had new worm nests (with a total of 8 new nests) at 12 months. At 36 months, 2 additional individuals in the IA arm were observed to have 1 new worm nest each. IDA recipients had no new adult worm nests detected at 6 or 12 months. However, 2 IDA recipients had a total of 3 new worm nests at 24 months and 2 additional IDA recipients each had 1 additional new worm nest at 36 months.

### Retreatment of Microfilaria-positive Individuals at 24 Months

Fourteen individuals treated with IDA at baseline were Mf positive at 24 months, 9 of whom were positive at 12 months (Table 2, Supplementary Figure 2). The Mf-positive individuals at 24 months were retreated with IDA and all but 1 individual completely cleared microfilariae. Of note, the baseline geometric mean Mf counts for individuals who were Mf positive at 24 months (277 Mf/mL) versus those who were Mf negative at baseline (175 Mf/mL) were similar (*P* = .14).

### Adverse Events After Treatment

Ninety-one of 95 individuals treated (96%) were evaluated for AEs 24 hours posttreatment. Of participants in the IDA and IA arms 47% and 40% had AEs after treatment, respectively (Table 4). No severe (grade 3) or serious AEs were observed. The frequency of mild, subjective AEs (grade 1) was similar between the 2 treatment arms, with headache, joint pain, fatigue, and nausea being the most common symptoms (Supplementary Table 1). Grade 2 AEs occurred in 5 participants (12%) after IDA and in 1 participant (2%) after IA (Table 4) (*P* = .07). The odds of a participant experiencing a grade 2 AE increased by 23% for each incremental increase of 100 Mf/mL count (odds ratio, 1.23; 95% CI, 1.09–1.43; *P* = .04).

**Table 4.** Adverse Events Following Treatment of Lymphatic Filariasis

	IDA (n = 43)	IA (n = 48)
At least 1 AE n (%)	20 (47)	19 (40)
Individuals with ≥2 AEs	14 (33)	6 (13)
Severe or serious AEs	0	0
Fever ≥37.5°C^a^	7 (17)	6 (12)
Hemodynamic changes^b^	4 (10)	2 (4)
Overall grade 1 AEs (subjective)^c^	20 (47)	19 (40)
Overall grade 2 AEs (subjective)^c^	5 (12)	1 (2)
Grade 2 AEs by symptoms		
Fatigue	2 (5)	0
Nausea/vomiting	1 (2)	1 (2)
Muscle ache	0	1 (2)
Joint pain	1 (2)	0
Eye swelling	1 (2)	0
Rash	1 (2)	0
Lightheadedness	0	1 (2)
Dyspnea	1 (2)	

Abbreviations: AE, adverse event; IA, ivermectin plus albendazole; IDA, ivermectin plus diethylcarbamazine plus albendazole.

^a^The number of individuals with temperatures ≥37.5°C based on axillary temperature. The highest temperature recorded posttreatment was 38.4°C.

^b^Defined as a change in blood pressure of 30 mm Hg systolic or 20 mm Hg diastolic compared with the pretreatment recording. One subject in the IA arm developed transient hypertension (170/110) and another had a decrease in systolic blood pressure not considered to be hypotensive (100/60). One subject treated with IDA also developed transient hypertension (160/110) and 3 subjects had decreases in systolic blood pressure, but without meeting criteria for hypotension. None of these changes required medical intervention (all were grade 1).

^c^Only grade 2 AEs are listed by symptoms. With respect to specific grade 2 AEs, 1 participant in the IA treatment arm had 3 different grade 2 AEs and 2 participants in the IDA treatment arm had 2 different grade 2 AEs.

## Discussion

Results from this study show that a single dose of IDA was more effective for clearing *W. bancrofti* microfilaremia at 6 and 12 months, and at least as effective as 2 annual doses of IA at 24 months posttreatment. By 36 months, however, 3 annual treatments of IA were superior in complete clearance of Mf compared with a single dose of IDA. The overall greater cumulative reduction in Mf in the first 2 years with a single dose of IDA compared with 2 doses of IA shows better efficacy of IDA. This increased efficacy is likely related to the macrofilaricidal effect of DEC, as previously described [15, 16] and documented in the current study, with increased inactivation of adult worms and reduction in CFA levels compared with IA. Many people treated with IDA who had sustained clearance of Mf after treatment had persistent filarial antigenemia. This suggests that most worms not killed by the IDA regimen were sterilized, which is consistent with data from other studies [3, 4]. As IA also reduced worm nests by 53% at 12 months posttreatment, this suggests that ALB alone has some macrofilaricidal activity [19]. However, additional doses of IA at 12 and 24 months had little additional effects on clearing worm nests that survived the first treatment, suggesting some worms were unresponsive to ALB. Similarly, retreatment of Mf-positive individuals with IDA in the IDA arm at 24 months had little effect on further clearance of worm nests or reduction in CFA levels. This failure to inactivate some worm nests with repeated DEC treatment has been previously observed [20], especially in younger individuals [21] and in high transmission areas [22], suggesting that younger worms may be less susceptible to treatment.

There are several limitations to this study. Most participants in the study were males (see explanation in Methods). Due to logistical reasons, we were unable to reach our desired sample size; however, posttreatment follow-up had fewer dropouts than anticipated. Another limitation of the study was the retreatment of Mf-positive individuals at 24 months following a single dose of triple-drug therapy. This was not in the original protocol; however, this was requested by the Ivorian Institutional Review Board. This did not affect our primary endpoint of Mf clearance at 36 months. We considered all persons who were Mf positive 24 months after IDA treatment to have failed to clear Mf at 36 months, because they were unlikely to have cleared Mf during the subsequent year without retreatment. There were fewer individuals in the IDA arm because some individuals were excluded after being assigned to this arm and subsequently identified with onchocerciasis.

Moderate (grade 2) AEs were slightly more frequent after the first IDA treatment than after IA, but all AEs resolved within 2 to 3 days, which is consistent with other studies of IDA [23]_._

This study confirms that single-dose treatment with IDA is well tolerated and more efficacious against *W. bancrofti* larvae and adults than IA and comparable to 2 rounds of IA. The failure of IDA to sustain clearance of Mf at 36 months, as was observed in PNG, may be related to reinfection, as suggested by the presence of new worm nests. This study is the first demonstration of IDA efficacy in Africa, and our results support mathematical models that suggest that annual treatment with IDA could decrease the number of rounds of MDA needed to achieve elimination [24, 25], which would be extremely valuable in logistically difficult areas. Currently, IDA cannot be used in areas coendemic with onchocerciasis, due to the potential effects of DEC on those with high onchocercial Mf levels. However, IDA could be used in LF “hot spots” that are coendemic with onchocerciasis, if Mf levels can be reduced by pretreatment with IVM prior to IDA. Studies are currently ongoing to determine if this is feasible.

## Supplementary Data

Supplementary materials are available at *Clinical Infectious Diseases* online. Consisting of data provided by the authors to benefit the reader, the posted materials are not copyedited and are the sole responsibility of the authors, so questions or comments should be addressed to the corresponding author.

ciz1050_suppl_Supplementary_Information

## Notes

***Author contributions.*** All authors contributed substantially to this article. C. L. K., G. J. W., and B. G. K. were responsible for study design; and C. L. K., C. M. B., and B. A. performed literature searches. Data were collected by C. M. B., A. F. O., O. K., V. K. M., and B. A.; data were analyzed and interpreted by M. A., G. J. W., B. G. K., and C. L. K. The manuscript was written by C. M. B., A. F. O., M. A., B. A., G. J. W., B. G. K., and C. L. K. All authors approved the final draft.

***Acknowledgments.*** The authors gratefully acknowledge the participation and cooperation of the study participants, as well as the work of study investigators who provided essential help with technical issues, community engagement, and clinical aspects.

***Disclaimer.*** The funders had no role in the study design, data collection, data analysis, or interpretation of data; in writing of the manuscript; or the decision to submit for publication. The corresponding author had full access to all the data in the study and had final responsibility for the decision to publish.

***Financial support.*** This work was supported by the Bill & Melinda Gates Foundation (grant number OPPGH 5342).

***Potential conflicts of interest.*** G. J. W. and B. A. report grants from the Bill & Melinda Gates Foundation, outside the submitted work. C. L. K. reports grants from the Bill & Melinda Gates Foundation, National Institutes of Health, and the Veterans Affairs Administration, outside the submitted work. C. M. B. reports grants from the Bill & Melinda Gates Foundation, during the conduct of this study, and grants from the Bill & Melinda Gates Foundation, outside the submitted work. All other authors report no potential conflicts. All authors have submitted the ICMJE Form for Disclosure of Potential Conflicts of Interest. Conflicts that the editors consider relevant to the content of the manuscript have been disclosed.
