# Supplementary material for: Efficacy and Safety of a Single Dose of Ivermectin, Diethylcarbamazine, and Albendazole for Treatment of Lymphatic Filariasis in Côte d’Ivoire: An Open-label Randomized Controlled Trial
Source: Clin Infect Dis. 2019 Oct 23;71(7):e68–75. doi: 10.1093/cid/ciz1050 (PMC7583415; doi:10.1093/cid/ciz1050)
Supplement: ciz1050_suppl_Supplementary_Information [file ciz1050_suppl_supplementary_information.docx]

**Supplemental table 1.** Comparison of subjective adverse events between treatment groups.*

|  | IA arm (N=48) | |  | IDA arm (N=43) | |
| --- | --- | --- | --- | --- | --- |
|  | Number individuals affected | (%) |  | Number individual affected | (%) |
| Headache | 7 | 16.7 |  | 8 | 18.6 |
| Joint pain | 4 | 8.3 |  | 8 | 18.6 |
| Fever | 5 | 10.4 |  | 5 | 11.6 |
| Fatigue | 5 | 10.4 |  | 5 | 11.6 |
| Nausea/vomiting | 4 | 8.3 |  | 3 | 7.0 |
| Rash/itch | 2 | 4.2 |  | 4 | 9.5 |
| Muscle ache | 3 | 6.3 |  | 2 | 4.7 |
| Cough | 1 | 2.1 |  | 3 | 7.0 |
| Lightheaded/dizzy | 2 | 4.2 |  | 2 | 4.7 |
| Shortness of breath | 1 | 2.1 |  | 3 | 7.0 |
| Diarrhea | 2 | 4.2 |  | 0 | 0.0 |
| abdominal pain | 0 | 0.0 |  | 1 | 2.3 |
| Palpitations | 1 | 2.1 |  | 0 | 0.0 |
| Dark urine | 0 | 0.0 |  | 1 | 2.3 |

* Grade 1 subject adverse events that occurred following

**Supplemental Figure 1**. The number of new worm nests detected at 6, 12, 24, and 36 months post-treatment.

**Supplemental Figure 2.** The effect of re-treatment with IDA on Mf counts at 36 months in individuals who were Mf-positive 24 months after IDA treatment and re-treated at that time (N=14). Mf counts at baseline and 12, 24, and 36 months were plotted on a logarithmic scale. Arrows indicate timing of IDA administration.
